# Undocumented Migrants’ Experiences of a Recovery-Oriented Group Intervention and Its Impact on Their Mental Well-Being: A Qualitative Study

**DOI:** 10.3390/ijerph22111617

**Published:** 2025-10-23

**Authors:** Zoë Nieuwhof, Maaike Kooiman, Willem F. Scholte, Marianne Reddingius, Martha Teijema

**Affiliations:** 1 Doctors of the World, 1018 DP Amsterdam, The Netherlands; 2 Parnassia Groep Rijnmond, 3172 AA Poortugaal, The Netherlands; 3 Human Rights Initiatives, 1013 RR Amsterdam, The Netherlands; 4 Laguna Collective, 1074 EG Amsterdam, The Netherlands; 5 Department of Adult Psychiatry, Amsterdam University Medical Center, University of Amsterdam, 1105 AZ Amsterdam, The Netherlands; 6 Antares Foundation, 1074 EG Amsterdam, The Netherlands; 7 Stichting Loa Foundation, 1017 ZC Amsterdam, The Netherlands

**Keywords:** personal recovery, undocumented migrants, mental health, METS, CHIME, refugees, asylum seekers

## Abstract

The Method for the Empowerment of Trauma Survivors (METS) is a recovery-oriented group intervention tailored to refugees and asylum seekers who experienced traumatic events. This study explores how undocumented migrants in the Netherlands experience participation in METS, how these experiences relate to changes in their mental well-being, and which aspects of the intervention participants find most valuable. A qualitative case study was conducted involving in-depth, individual interviews with undocumented migrants who participated in METS. Interviews focused on participants’ experiences with the intervention and perceived changes in mental well-being. Five main themes emerged: connectedness, group dynamics, personal development, emotional well-being, and practical aspects. Changes in mental well-being were often subtle, difficult to articulate, and in some cases temporary. While some participants reported positive developments, many continued to face significant challenges and did not experience notable improvement. No participants reported negative outcomes as a result of participation. METS appears suitable for a heterogeneous population with diverse cultural, religious, and educational backgrounds. Reported benefits often related to group participation rather than METS-specific characteristics. Future research is warranted to further explore whether METS is a valuable addition to existing transdiagnostic psychosocial group interventions.

## 1. Introduction

In 2024, 32,175 people applied for asylum in the Netherlands, for the first time. In that year, 31,120 asylum seekers were granted a temporary residence permit [1]. In general, those granted asylum gain refugee status and a residence permit, while individuals whose applications are denied do not receive this permit. Those who remain in the country without documentation are referred to as “undocumented migrants”. This group also encompasses individuals who never submitted an asylum application.

Research shows that refugees and asylum seekers are at a higher risk of developing mental health symptoms compared to the general population [2]. When comparing statistics on refugees (those who have received a residence permit) and asylum seekers, self-reported symptoms of PTSD, depression, and anxiety are more prevalent among asylum seekers [3,4]. This points towards a negative impact of an undocumented status. A study by Vollebregt et al. [5] focused on help-seeking undocumented migrants in the Netherlands and found that 85% scored above the clinical threshold on the Self-Reporting Questionnaire 20 (SRQ-20), indicating the likelihood of the existence of mental health problems in a vast majority.

It is estimated that between 23,000 and 58,000 individuals resided in the Netherlands without legal documentation during the period 2017–2018 [6]. However, quantifying the undocumented migrant population is challenging. The lack of documentation not only complicates population estimates but also limits the ability to satisfy basic needs, making it nearly impossible to find legal housing, (volunteer) jobs, or access to necessary healthcare. Likewise, it complicates the accessibility of required psychological treatment. Several barriers hinder access to care, including cultural and language barriers, limited awareness of available services, fear of deportation, frequent relocations, and (self-) stigma. Additionally, mental health professionals may be hesitant to provide treatment to undocumented migrants [7].

When treatment is provided, traumatized asylum seekers and refugees seem to benefit less than other traumatized control groups (patients with profession-related trauma such as police officers and military veterans), treatment response can be limited, and PTSD symptom severity often remains high [8]. Treatment often focuses on pre-migration traumatic events, which have been shown to have a direct relation with levels of anxiety and depression [9]. However, research has also emphasized the importance of the post-migratory context, showing how post-migration stress is related to poorer mental health and arguing for the promotion of resilience and stress-reduction after migration [10,11]. It is argued that interventions focusing on quality of life might be useful for refugees and asylum seekers [8]. In dealing with post-migratory problems, research shows self-efficacy is significantly related to lower psychological distress and higher subjective well-being [12].

Since trauma therapy (if available to these patients at all) is not always successful, and in order to take into consideration the influence of post migration stressors on the one hand and self-efficacy on the other, a recovery-oriented group intervention method called the Method for the Empowerment of Trauma Survivors (METS) was developed during a collaborative project of seven European refugee rehabilitation centers. This method is based around the concept of personal recovery, defined by Anthony [13] as “a deeply personal, unique process of changing one’s attitudes, values, feelings, goals, skills, and/or roles. It is a way of living a satisfying, hopeful, and contributing life even with limitations caused by illness. Recovery involves the development of new meaning and purpose in one’s life as one grows beyond the catastrophic effects of mental illness”.

To operationalize the concept of personal recovery for the development of METS, the framework as developed by Leamy et al. [14] was used. This conceptual framework is based on five identified pillars for personal recovery: Connectedness, Hope, Identity, Meaning, and Empowerment, collectively referred to as the CHIME framework. Designed to guide recovery-oriented research and practice, the CHIME framework has primarily been used to assess existing interventions and instruments [15,16,17,18]. While it was applied predominantly among white populations [19], Leamy et al. [14] themselves stated that more research on the applicability of CHIME for ethnic minorities was needed. Additionally, the framework has mostly been employed in studies focusing on individuals already involved with secondary specialist services [19].

Besides METS, several other low-intensity transdiagnostic psychosocial interventions have been developed within the field of global mental health. These interventions are typically designed to be delivered by non-specialized healthcare workers in low- and middle-income countries. Notable examples of these interventions are Self-Help Plus (SH+), Problem Management Plus (PM+), and Common Elements Treatment Approach (CETA) [20,21,22].

METS was first evaluated by Van Heemstra et al. [23] (In this study Van Heemstra et al. [23] refer to the METS method as 7ROSES. This term is also used to denote the METS method and refers to the seven topics addressed within the method.), using two quantitative instruments: the General Self-Efficacy Scale (GSES) to measure changes in the level of self-efficacy, and the Brief Symptom Inventory (BSI) to evaluate changes in general mental health. Previous research had demonstrated that self-efficacy helps to reduce psychopathology in refugees [12] and enhances tolerance for trauma-related stress [24]. In their study, Van Heemstra et al. [23] conclude that METS could slightly improve both general mental health and self-efficacy in refugees experiencing psychopathology.

The current evaluation builds on this research, with a specific focus on undocumented migrants. This focus is informed by the findings suggesting that individuals without the security of a residence permit have a different post-migratory experience than refugees, leading to different psychological challenges.

This study evaluates the implementation of METS among undocumented migrants residing in the Netherlands who are not (yet) receiving (trauma) therapy. It aims to explore how participants experience METS and how these experiences relate to changes in personal well-being. Additionally, the study examines which aspects of METS are particularly valued by its participants and how this can be compared to other group interventions.

## 2. Materials and Methods

### 2.1. Design and Setting

A qualitative case study was conducted using semi-structured in-depth individual interviews using an interview topic guide, which can be accessed from the Supplementary Materials. We chose this qualitative methodology because it facilitates the obtaining of comprehensive insight into the participants’ experience and helps identify which specific aspects of the intervention they value [25]. Interviews took place at the office of Doctors of the World, an NGO in Amsterdam that provides support for and direct medical care to undocumented migrants. The interviews took place in the same room where the participants attended the METS meetings, aiming to provide a familiar and safe space. Due to feasibility reasons, one interview with a METS facilitator took place via an online video call.

### 2.2. Intervention

METS is not a substitute for trauma therapy. It is a group intervention aiming to raise awareness of both the meaning and importance of the core elements of personal recovery. METS explores and practices strategies with participants to focus on their personal recovery. Next to the five pillars of the CHIME framework, METS focuses on two additional themes: recognition and safety (After the performance of this study, during further development of METS, these elements were integrated in the meetings dedicated to the CHIME pillars, reducing the number of meetings to five.). Each theme is addressed in a dedicated session, while the complete series is preceded by an introductory meeting and followed by a concluding meeting, coming to a total of nine meetings within the intervention.

As a rule of thumb, METS has a minimum of four and a maximum of ten participants. Meetings took place on a weekly basis and lasted two to three hours. Meetings were facilitated by volunteers who usually had a background in psychology. Each meeting started with an introduction about the topic to inform participants about the goal of the meeting, followed by a group dialogue. The remainder of the meetings consisted of different exercises on the topic which were alternated by energizing activities.

### 2.3. Participants

Participants for this study were recruited through their participation in one of two previous rounds of METS provided by Doctors of the World in Amsterdam. The organization offers METS to undocumented migrants if they ask for psychosocial support, either on their own accord or through other support organizations. Participants did not receive any mental healthcare besides supportive counseling from a volunteering psychologist of Doctors of the World during METS. In this setting, sufficient proficiency in the English language was a condition for joining METS. Exclusion criteria for participation in the meetings were symptoms indicating possible psychosis and a general behavior pattern which would likely disturb group interaction. There was no participation fee, and participation in this study was not a condition for being allowed to attend the meetings.

Purposive sampling was used based on the criterion that participants had attended at least two METS meetings. However, not all individuals meeting this criterion could be included, due to participants’ availability and their willingness to participate in the study. As a result, ten interviews were conducted with individuals who participated in METS, as well as two interviews with facilitators experienced in providing METS. Inclusion of the perspectives of the facilitators broadened the scope of our data, as they could evaluate METS from a different point of view. Their experience enabled them to compare different METS groups and to consider its effects on the participants from an outsider perspective.

### 2.4. Data Collection

Three members of the research team conducted the semi-structured interviews, working in pairs. Two of the interviewers were sociologists and the third was a medical doctor working in psychiatry. Apart from their role in conducting the research, the interviewers were not involved with the METS program. One interviewer acted as the main interviewer while the other interviewer mainly observed and asked additional questions if deemed valuable. Interviews were structured using an interview guide based on the seven topics of the METS meetings. First, participants were asked general questions about their life and their thoughts on METS. Next, specific questions about the content of METS meetings were asked. The semi-structured nature of the interviews allowed the interviewer to personalize the questions and explore specific responses further.

A separate interview guide (see Supplementary Materials) was used for interviewing the facilitators. This one included questions on their experiences facilitating METS, what they thought were strengths and limitations of the method, and if they had noticed changes in participants along the way. One interview with a facilitator was carried out in Dutch, and all other interviews were conducted in English

Prior to conducting the interview, all the participants received a written and verbal clarification about the study and their participation. They were informed about their rights and guaranteed anonymity, after which written consent for participating and recording of the interview was obtained. Moreover, it was explicitly mentioned that their responses would not change their relationship with Doctors of the World in any way.

### 2.5. Data Analysis

All interviews were recorded and anonymously transcribed verbatim in the same language as the interview and were analyzed using an inductive approach to qualitative content analysis. Two researchers (ZN and MK) independently read and re-read two interviews forming a general impression of the data and individually started open coding. After applying open inductive coding to these first interviews a list of codes was generated. These codes were assessed, and by discussing our first interpretations, a consensus was reached on a coding scheme for the interviews. After this, all other interviews were coded. MK and ZN discussed the data multiple times with MT to include new thoughts after which MK and ZN moved to axial coding. In this process, concepts arose defining the themes and subthemes of the data. Data analysis was performed using ATLAS.Ti 22 qualitative analysis software.

### 2.6. Ethical Considerations

Ethical clearance was obtained from the medical research ethics committee from the Amsterdam University Medical Center, which judged that the Medical Research Involving Human Subjects Act [26] did not apply to this study and granted exemption to request official ethical approval.

## 3. Results

During the interviews, participants reflected on their experience with METS and the potential changes in personal well-being that might be associated with it. The experiences and changes can be categorized in the following themes: connectedness, group dynamics, personal development, emotional well-being, and practical aspects. The data will be presented according to these themes. Experienced changes in mental well-being were often subtle, for many hard to put into words, and sometimes only experienced temporarily. There were multiple examples of participants who did not experience any notable change and instead mentioned ongoing challenges (that remained beyond their control). However, no results were found suggesting that participation in METS led to any negative change in mental well-being. Table 1 shows the characteristics of the METS participants.

### 3.1. Connectedness

The most prominent theme arising from the data is the theme connectedness. It can be divided into four subthemes: connecting with others, opening up, feeling supported, and connecting with group members.

#### 3.1.1. Connecting with Others

Before participating in METS, many participants expressed feeling lonely and found it difficult to talk with others or to ask for help. As one of the participants mentioned:

*‘Before my life was full of loneliness. I was not going out with people, I was always staying inside and I was not socializing. Things I enjoy, but didn’t do.’*

(participant 2)

For some participants, METS seemed to have had a positive influence on their connection with others. For example, some participants mentioned (re)connecting with people during or after METS:

*‘I have friends from Iran and for a long time I wasn’t in contact with my friends. I made contact with them again and it was good. Because they understand I’m depressed because of this situation. And it is a good start for me, it is a good start.’ *

(participant 7)

Not all participants experienced improvements in their connection with others during or after METS. Participant 3 said:

*‘My connection with other people, no it is not a big difference it is the same.’*

(participant 3)

At the same time, this participant did have the desire to connect with others, which is illustrated by the following quote:

*‘It’s very hard when I see myself here lonely. I remember being in my city and a lot of people knew me. Now it feels very bad. I’m like a stranger, nobody knows me. In the shelter I live in now people don’t know anything about me. It is like I want to say who I am. Talk about everything: about my education, about my job, about my skills. You know about everything. But I cannot and it feels very bad.’*

(participant 3)

Furthermore, positive changes in connectedness were sometimes only temporary. One of the participants described how they connected with other participants during the meetings, and what this finally resulted in:

*‘We were just friends for the group. When everything was finished everybody was going their own way.’*

(participant 9)

#### 3.1.2. Opening Up

In addition to (re)connecting with others, positive changes were found regarding participants’ ability to open up to others and ask for help. Some participants described how after METS it was easier for them to talk to others or to ask for help, a topic which is also explicitly discussed during METS. One participant said:

*‘It was one of my biggest problems before the workshop. I had a lot of problems, but I didn’t want to ask for help. Now I try after that [the workshop]. I don’t want to say it solved it totally, but I try to ask for help more than before.’ *

(participant 3)

Another participant said:

*‘Now when I have something I open up easily. Yeah I can open up a little bit, which was not before. The METS has helped me to make contact with others. When I’m in the shelter I read on the board what activities there are. Now I will say ‘let’s try it’, but before I would say it is not for me. I used to carry my own problems thinking it is me alone, but now I know there are other people who are going through what I’m going through.’*

(participant 6)

One participant described finding it easier to ask for help, but believed this should be limited to seeking help from professionals:

*‘But through this workshop I found out it is very important to tell professionals, not everyone, about your problems.’*

(participant 2)

#### 3.1.3. Feeling Supported

The data show some explicit information about how participants experienced the relationship with the facilitators of the METS meetings. Participants especially valued the support they received from the facilitators and other people working at Doctors of the World. One participant said:

*‘I have this feeling that now there are some people here who care about me and support me. Yeah, it is very important to me and it helps a lot.’*

(participant 3)

Another participant explicitly mentioned feeling appreciation towards the facilitators for voluntarily helping vulnerable people in society.

#### 3.1.4. Connection with Group Members

Another subtheme related to connectedness concerns the relations between participants. Relations were experienced as both positive and negative, although a positive evaluation was more common. One participant discussed how group members enjoyed being with one another:

*‘I also liked the other students. They respect each other (…). This brought more happiness into my life because I met new people with different views of life.’*

(participant 2)

Yet, another participant mentioned having difficulty connecting with the other group members:

*‘Unfortunately I couldn’t make a connection with the group. I felt they are totally different.’*

(participant 3)

In addition, one participant talked in a frustrated manner about how other group members were not actively participating in the meetings:

*‘Really, I do not know why they come here and spend time for nothing. (...)If you don’t know why you need this, why do you come here and spend your time?’*

(participant 7)

### 3.2. Group Dynamics

Closely related to the subtheme ‘connection with group members’ is the broader theme of group dynamics. Group dynamics appear to have influenced participants’ experiences of METS and may have been shaped by connection as experienced among group members. One facilitator reflected on the group dynamics when stating:

*‘...in a kind of beautiful and mysterious way, all participants put their negativity together and converted it into strength and uplifted one another.’*

(facilitator 1)

Additionally, group dynamics may have been influenced by participants’ motivation for METS. Both facilitators and participants mentioned the importance of motivation for the fruitfulness and value of participation in the meetings. One participant mentioned:

*‘I knew I needed help and because of the fact that I was searching for help and got something that was helpful, it became helpful to me.’*

They also said:

*‘So we in the METS should be united because of the fact that we need and want help and that was what was lacking. And if that is lacking it doesn’t work in my opinion.’*

Additionally, facilitator 2 mentioned that when people regularly do not show up, the intervention does not really work:

*‘We had a group in which not many people showed up. Sometimes we were only two or three people and then you notice it just doesn’t really work.’*

Finally, the educational level of the participants may also be linked to the way METS is experienced and may influence group dynamics. One of the facilitators answered the following when asked about what personal characteristics of the participants may have influenced their takeaway from METS:

*‘Education. Because [when] someone speaks English [it] does not mean they know how to analyze a question logically and define the rightful answers.’*

(facilitator 1)

Referring to both a barrier and discrepancy when it comes to understanding the questions and topics that are introduced during the meetings in METS.

### 3.3. Personal Development

A few participants described improvements in their self-esteem and confidence, things that could be referred to as personal development. One participant said:

*‘The workshop taught me a lot of things. Especially for me to add more value to myself. Because before the workshop I was thinking I am worthless and valueless to society. Thinking I am inferior to society. But the METS taught me to add more value to myself.’*

(participant 2)

Another participant discussed how they were invited by a University to talk about their lives and how they would not have felt comfortable doing this before they participated in METS:

*‘I was invited to go to the University of Amsterdam to talk about myself, which I could not have done if I hadn’t attended the workshop. Because of the workshop now I can at least try.’* They also added *‘So I’m proud.. I got that from the workshop, just this small self-confidence I have. I have never been confident before.’*

(participant 6)

A positive impact on self-esteem did not apply for all participants. Some participants discussed how they felt like they had lost their identity and how this had not changed after METS:

*‘I lost everything I had. My connections with my friends, my family and my job. All these things make my identity. Now I feel I lost it and it’s difficult.’*

(participant 3)

### 3.4. Emotional Well-Being

All participants described having mental health complaints before starting METS. Participants talked about feeling isolated, feeling sad, having trouble sleeping, and having nightmares. Three of the participants mentioned that these complaints included suicidal ideations. For example, one participant said:

*‘A lot of things are going on in your mind, coping with the things I experienced back home, before coming. So a lot of nightmares, a lot of things, lack of concentration and suicide was becoming an option.’*

(participant 5)

Because participants often experienced these mental health complaints, facilitators sometimes decided to do mindfulness exercises with the participants throughout METS. Many participants mentioned how they liked these exercises and some of the participants still used them on a regular basis. One participant mentioned they wished there were more practical exercises like this in METS:

*‘Do you remember learning the techniques with breathing to feel calm again?’*

(Interviewer)

*‘Yes and it helps a lot.’*

(Participant 3)

*‘Do you do it on your own?’*

(Interviewer)

*‘Yes every night’*

(Participant 3)

*‘Oh really. Does it help you sleep?’*

(Interviewer)

*‘Yes it’s very good. I wish there were more practical techniques like this.’*

(Participant 3)

Apart from finding these mindfulness exercises helpful, some participants experienced improvements in their mental health after METS. One participant explicitly mentioned they no longer had suicidal ideations after METS:

*‘This workshop taught me about hope and thinking positive. Now, that negative thought, that always came to my heart, to kill myself, is not anymore.’*

(participant 2)

Another participant talked about how in the period after METS they experienced laughter for the first time. In response to the question if any big life events happened for the participant after completion, they said:

*‘I told you I was laughing deeply. Before I didn’t have any experience like that. I fell on the ground laughing.’*

(participant 7)

Although some participants talked about improvements in their emotional well-being, this was not the case for everyone. One of the participants talked about only experiencing temporary change:

*‘The biggest problem is the change was temporary. I had a very good feeling during the workshop and after, but it was only for a few days. And after a few days again I had those black things in my head.’*

(participant 3)

Furthermore, despite following METS, many participants still struggled with their mental health, often related to the fact they were living undocumented. When interviewing the participants, the interviewers could sometimes feel their sadness. One participant said:

*‘The METS is good, there is nothing wrong with the METS, but the METS cannot, even the psychologist cannot take everything away from my mind from my past. They cannot take everything in me because I passed through many difficult things in life.’* They also said: *‘Yeah but my life now is not okay, but it is just … the nightmare is still there and the think is still there and my procedure is also still there.’*

(participant 4)

Another participant said something indicating they were glad they were getting older and closer to death:

*‘We don’t know about tomorrow or how my life will go or something, but like if I turn seventy or eighty years old, most of my life I have spent already.’*

(participant 1)

In addition, participants’ precarious living situation due to their undocumented status was sometimes mentioned as a cause for problems with concentration during participation in METS. For example:

*‘Unfortunately the mind was not the same when I came here after going to the attorney. Fifty percent of my mind was…’ Interviewer: it was with the problems. Participant 1: yeah so really I can’t concentrate.’*

(participant 1)

No indications were found that participants experienced negative changes in their emotional well-being after participating in METS.

### 3.5. Practical

Another theme that emerged was METS as a place to help participants find their way in a new living environment, adapt to a new culture, and learn what their rights are. Besides having to deal with the struggles of living without documents, participants also have to deal with the challenge of living in a new country and culture. One participant mentioned how in Dutch culture, you need to ask questions if you want to get help, and how they did not know about this before. They also mentioned not knowing what their rights were:

*‘I came from a collectivist culture with a dictatorship government and I didn’t know about 21st century freedom and democracy. I didn’t know about my rights here and nobody explained it to me. Because here it basically looks like this: if you are asking questions people have answers for you, but if you don’t ask questions nobody gives you an answer.’*

(participant 7)

One of the facilitators mentioned that METS may help participants to get acquainted with Dutch culture. Facilitators can for example explain things about Dutch culture to participants. As one participant stated:

*‘Here in Holland everybody asks you ‘why did you come?’. The workshop helped me to know that maybe it is a common question for all the Dutch people, but in the beginning I would think it is an insult. However, when someone brought up the topic they explained ‘no, sometimes they ask you why you’re here for you to get help’, but in the beginning I didn’t take it like that. So now I came to learn that some questions are there to help me out.’*

(participant 6)

The same participant also discussed how METS helped them to find their way in a new city:

*‘Since I came to Amsterdam so many things have changed. I didn’t know anything. Although I was in Holland, I didn’t know what the metro was. I didn’t know how to use the tram. I didn’t know where to go. The first time I came here I was thinking: how am I going to fit in this society? Now I slowly learned how to fit in situations. If I did not attend this workshop and wouldn’t have met other people. Whenever I would move to another place. Whenever I’d go to a new situation I would get anxiety, but now when I move to a new place, now I know how to do things. The little knowledge I got from here, I know how to find my way in the city now, without hurting myself.’ (participant 6) Some also mentioned they learned about their rights: ‘I learned everybody in Holland has the right to medication and treatment. I also learned that if you have any problems you have numbers to call. That is what I learned in the workshop.’*

(participant 6)

## 4. Discussion

Our research included a variety of people participating in METS, each bringing unique cultural, personal, emotional, religious, and educational backgrounds, living in complex and uncertain circumstances, and often having experienced trauma. The findings suggest that these circumstances influence participants’ experience of METS and that achieving the intended outcomes, as related to the CHIME pillars, is therefore complex. Barriers to full engagement in METS were identified across various themes, including differences in educational levels and uncertainties related to legal procedures. Despite these challenges and the diversity of backgrounds, the results demonstrate an overall positive experience of METS. For most participants in the study, the topics addressed during the meetings resonated and were meaningful to a certain extent. This experience was both shared and endorsed by the facilitators. These findings suggest that METS may be suitable for a culturally diverse group. Themes derived from the CHIME framework appeared to be valuable and relevant for the undocumented migrants who participated, indicating potential applicability across a heterogeneous group. This finding aligns with Brijnath [19], who examined the applicability of the CHIME framework in Anglo–Australians and Indian–Australians, and found it applicable to both groups.

In assessing the experience of METS, the theme of connectedness—entailing participants’ connection with others, with the group, and with the trainers—emerged as the most prominent. This theme corresponds with the first pillar of the CHIME framework and aims to promote social connectedness as it is associated with improved health outcomes, whilst on the contrary, social isolation is associated with mental illness [27,28]. Several participants reported explicit improvements in their social connections. As one participant mentioned that their connection with other group members only lasted for the duration of the workshop, we suggest that at the end of METS, an attempt could be made for the group to continue meeting each other, for example, by facilitating an app group or a fixed appointment time in nearby social facilities. Another approach to enhancing group cohesion could involve the creation of more coherent groups, e.g., by selecting participants with similar educational backgrounds or from the same country. However, this may present challenges, as one of the issues faced by undocumented migrants is the difficulty in finding like-minded individuals.

The experienced changes in mental well-being related to participation in METS varied in intensity and duration. Some participants reported no change in mental well-being, while others noted subtle yet meaningful changes, such as improved self-esteem or no longer experiencing suicidal ideations after attending METS. These reported effects were diverse but generally positive. No clear relationship was found between the number of METS meetings attended and the reported experiences. Other underlying factors may have influenced both attendance and post-intervention outcomes. Although these findings make it difficult to directly attribute improvements to METS in particular, the method may have played a facilitating role.

This research found that some improvements in mental well-being may relate to group participation in general rather than to METS-specific characteristics. There is ample literature showing benefits of group-based mental health programs. An extensive literature review by Dickson and Bangpan [29] found that these benefits include ‘the importance of the group as a resource and a source of support’, reducing social isolation, and promoting social cohesion by connection with others in similar circumstances. Similar results were found in our study. Furthermore, several studies have shown that group cohesion (an element of group dynamics), the relationships within a group as a whole, or the group climate predicts treatment outcome [30,31,32]. Our study found that group dynamics, influenced by the various characteristics of participants and participants’ motivation to join METS, may influence the extent to which the group is a source of support.

### 4.1. Strengths and Limitations

To our knowledge this is the first study qualitatively evaluating a mental health group intervention for undocumented migrants—an often marginalized and underrepresented group in research—and providing insights into their experiences. Although it is often a challenge to engage undocumented migrants in research, we successfully conducted interviews with undocumented migrants who had participated in a group-based mental health program. We interviewed a heterogeneous group of people, enabling the exploration of a broad range of perspectives and experiences, which enriched the depth and nuance of the data. Participants reported feelings of safety and positive experiences with Doctors of the World, which contributed to the quality and depth of the interviews.

There are, however, several limitations to our study. First, as this is an uncontrolled study, changes experienced by METS participants over time may also have been brought about by other factors. Second, we could only interview a limited number of participants, depending on whom we could reach and who was willing to participate, which may have introduced self-selection bias and limited the representativeness of the sample. Third, the topic guide for the semi-structured interviews included questions that, in hindsight, may be considered leading. By explicitly addressing the various components of the METS framework, the interviews introduced specific themes rather than allowing them to emerge solely from participants’ spontaneous recollections or associations. This approach may have shaped how participants interpreted and responded to the questions, potentially influencing the content and structure of their answers. Fourth, findings from the interviews only give insights into what people say and not what they do. The researchers sometimes felt participants’ responses were socially desirable. Fifth, interviews with all METS participants were conducted in English, which was neither the participants’ nor the interviewers’ first language. English language skills were different for all participants, meaning a language barrier may have influenced participants’ responses to questions. Furthermore, since proficiency in English was a requirement for participation in METS, the study sample may not be representative of undocumented migrants in general. Finally, some participants were interviewed months after completing all meetings, influencing what they were able to recall.

### 4.2. Future Research

For future research on the impact of METS, studies should also include other refugee subgroups and settings (among other humanitarian crisis settings), such as asylum seekers and migrants in refugee camps. A useful next step would be the incorporation of quantitative methods which would allow randomized controlled trials. However, the use of quantitative outcome measures in non-Western populations is complicated, and currently, no validated outcome measure to evaluate METS exists. More qualitative research could feed into the development of such an outcome measure.

## 5. Conclusions

In this study on the experiences of METS for undocumented migrants residing in the Netherlands, the intervention appeared to be well-suited for a heterogeneous population with diverse cultural, religious, and educational backgrounds. This supports the applicability and relevance of METS in intercultural and transcultural contexts. The extent to which participants reported improvements in mental well-being varied, and certain changes appeared to stem more from group participation rather than METS-specific characteristics. Given its specific focus on refugees and its emphasis on participants’ personal recovery and strengths, future research seems relevant to explore whether METS is a valuable addition to the existing transdiagnostic psychosocial group interventions.

## Figures and Tables

**Table 1:** Characteristics of METS participants.

Name	Gender	Birthdate	Land of Origin	In the Netherlands Since	Number of METS Meetings Attended
Participant 1	male	1969	Pakistan	2005	7
Participant 2	male	1986	Nigeria	2019	8
Participant 3	male	1982	Iran	2019	4
Participant 4	female	1995	Nigeria	2019	6
Participant 5	male	1973	Nigeria	2006	8
Participant 6	female	1975	Uganda	2018	8
Participant 7	male	1997	Iran	2019	5
Participant 8	male	1997	Nigeria	2018	4
Participant 9	male	1998	Mali	2017	4
Participant 10	male	1994	Guinee	2020	7

## Data Availability

The datasets (i.e., interview transcripts and coding framework) generated and analyzed during the current study are not publicly available due to confidentiality agreements and the inclusion of potentially identifiable participant information. However, anonymized excerpts and thematic matrices are available from the corresponding author on reasonable request.

## Supplementary Materials

The following supporting information can be downloaded at: https://www.mdpi.com/article/10.3390/ijerph22111617/s1, Topic list.

## Abbreviations

The following abbreviations are used in this manuscript:

CHIME	Connectedness, hope, identity, meaning, empowerment
METS	Method for the empowerment of trauma survivors

## References

[1] CBS, Centraal Bureau Voor de Statistiek (2025). Hoeveel Asielzoekers Komen Naar Nederland? (How Many Asylum Seekers Come to the NETHERLANDS?). https://www.cbs.nl/nl-nl/dossier/dossier-asiel-migratie-en-integratie/hoeveel-asielzoekers-komen-naar-nederland.

[2] Jorgenson K.C., Nilsson J.E. (2021). The relationship among trauma, acculturation, and mental health symptoms in Somali refugees. Couns. Psychologist.

[3] Gerritsen A.A., Bramsen I., Devillé W., van Willigen L.H., Hovens J.E., Van Der Ploeg H.M. (2006). Physical and mental health of Afghan, Iranian and Somali asylum seekers and refugees living in the Netherlands. Soc. Psychiatry Psychiatr. Epidemiol..

[4] Toar M., O’Brien K.K., Fahey T. (2009). Comparison of self-reported health & healthcare utilisation between asylum seekers and refugees: An observational study. BMC Public. Health.

[5] Vollebregt S.J.C., Scholte W.F., Hoogerbrugge A., Bolhuis K., Vermeulen J.M. (2022). Help-Seeking Undocumented Migrants in the Netherlands: Mental Health, Adverse Life Events, and Living Conditions. Cult. Med. Psychiatry.

[6] Van der Heijden P.G.M., Cruyff M.J.L.F., Engbersen G.B.M., van Gils G.H.C. (2020). Schattingen Onrechtmatig in Nederland Verblijvende Vreemdelingen 2017–2018 (Rapport; Estimates of Undocumented Migrants Residing in the Netherlands 2017–2018). WODC. https://repository.wodc.nl/bitstream/handle/20.500.12832/3010/2969-schattingen-onrechtmatig-in-Nederland-verblijvende-vreemdelingen-2017-2018-volledige-tekst.pdf?sequence=7&isAllowed=y.

[7] Pharos (2019). oegang Tot Zorg Voor Ongedocumenteerde Migranten: Wat Helpt om Zorg te Krijgen? (Access to Healthcare for Undocumented Migrants: What Helps to Obtain Care?). https://www.pharos.nl/kennisbank/toegang-tot-zorg-voor-ongedocumenteerde-migranten-wat-helpt-om-zorg-te-krijgen/.

[8] Ter Heide F.J.J., Smid G.E. (2015). Difficult to treat? A comparison of the effectiveness of treatment as usual in refugees and non-refugees. BJPsych Bull..

[9] Bhui K., Abdi A., Abdi M., Pereira S., Dualeh M., Robertson D., Ismail H. (2003). Traumatic events, migration characteristics and psychiatric symptoms among Somali refugees: Preliminary communication. Soc. Psychiatry Psychiatr. Epidemiol..

[10] Carswell K., Blackburn P., Barker C. (2011). The relationship between trauma, post-migration problems and the psychological well-being of refugees and asylum seekers. Int. J. Soc. Psychiatry.

[11] Kronick R. (2018). Mental health of refugees and asylum seekers: Assessment and intervention. Can. J. Psychiatry.

[12] Sulaiman-Hill C.M., Thompson S.C. (2013). Learning to fit in: An exploratory study of general perceived self-efficacy in selected refugee groups. J. Immigr. Minor. Health.

[13] Anthony W.A. (1993). Recovery from mental illness: The guiding vision of the mental health service system in the 1990s. Psychosoc. Rehabil. J..

[14] Leamy M., Bird V., Le Boutillier C., Williams J., Slade M. (2011). Conceptual framework for personal recovery in mental health: Systematic review and narrative synthesis. Br. J. Psychiatry.

[15] Penas P., Uriarte J.J., Gorbeña S., Moreno-Calvete M.C., Ridgway P., Iraurgi I. (2020). Psychometric adequacy of recovery enhancing environment (REE) measure: CHIME framework as a theory base for a recovery measure. Front. Psychiatry.

[16] Shanks V., Williams J., Leamy M., Bird V.J., Le Boutillier C., Slade M. (2013). Measures of personal recovery: A systematic review. Psychiatr. Serv..

[17] Slade M., Leamy M., Bacon F., Janosik M., Le Boutillier C., Williams J., Bird V. (2012). International differences in understanding recovery: Systematic review. Epidemiol. Psychiatr. Sci..

[18] Yung J.Y., Wong V., Ho G.W., Molassiotis A. (2021). Understanding the experiences of hikikomori through the lens of the CHIME framework: Connectedness, hope and optimism, identity, meaning in life, and empowerment; systematic review. BMC Psychol..

[19] Brijnath B. (2015). Applying the CHIME recovery framework in two culturally diverse Australian communities: Qualitative results. Int. J. Soc. Psychiatry.

[20] Dawson K.S., Bryant R.A., Harper M., Kuowei Tay A., Rahman A., Schafer A., van Ommeren M. (2015). Problem Management Plus (PM+): A WHO transdiagnostic psychological intervention for common mental health problems. World Psychiatry.

[21] Epping-Jordan J.E., Harris R., Brown F.L., Carswell K., Foley C., García-Moreno C., van Ommeren M. (2016). Self-Help Plus (SH+): A new WHO stress management package. World Psychiatry.

[22] Murray L.K., Dorsey S., Haroz E., Lee C., Alsiary M.M., Haydary A., Bolton P. (2014). A Common Elements Treatment Approach for Adult Mental Health Problems in Low- and Middle-Income Countries. Cogn. Behav. Pr..

[23] Van Heemstra H.E., Scholte W.F., Haagen J.F., Boelen P.A. (2019). 7ROSES, a transdiagnostic intervention for promoting self-efficacy in traumatized refugees: A first quantitative evaluation. Eur. J. Psychotraumatology.

[24] Morina N., Bryant R.A., Doolan E.L., Martin-Sölch C., Plichta M.M., Pfaltz M.C., Nickerson A. (2018). The impact of enhancing perceived self-efficacy in torture survivors. Depress. Anxiety.

[25] Peters S. (2010). Qualitative Research Methods in Mental Health. Evid. Based Ment. Health.

[26] CCMO, Central Committee on Research Involving Human Subjects, Medical Research Involving Human Subjects Act (WMO). https://english.ccmo.nl/investigators/legal-framework-for-medical-scientific-research/your-research-is-it-subject-to-the-wmo-or-not.

[27] Reivich K., Shatte A. (2002). The Resilience Factor: 7 Essential Skills for Overcoming Life’s Inevitable Obstacles.

[28] Townsend K.C., McWhirter B.T. (2005). Connectedness: A review of the literature with implications for counseling, assessment, and research. J. Couns. Dev..

[29] Dickson K., Bangpan M. (2018). What are the barriers to, and facilitators of, implementing and receiving MHPSS programmes delivered to populations affected by humanitarian emergencies? A qualitative evidence synthesis. Glob Ment Health.

[30] Burlingame G.M., McClendon D.T., Yang C. (2018). Cohesion in group therapy: A meta-analysis. Psychotherapy.

[31] Lindgren A., Barber J.P., Sandahl C. (2008). Alliance to the Group–as–a–Whole as a Predictor of Outcome in Psychodynamic Group Therapy. Int. J. Group. Psychother..

[32] Ogrodniczuk J.S., Piper W.E. (2003). The effect of group climate on outcome in two forms of short-term group therapy. Group. Dyn. Theory Res. Pract..

