# Supplementary material for: Undocumented Migrants’ Experiences of a Recovery-Oriented Group Intervention and Its Impact on Their Mental Well-Being: A Qualitative Study
Source: Int J Environ Res Public Health. 2025 Oct 23;22(11):1617. doi: 10.3390/ijerph22111617 (PMC12652161; doi:10.3390/ijerph22111617)
Supplement: Supplementary file 1 [file ijerph-22-01617-s001.zip › Evaluating the METS topic list semistructured interviews (1).pdf]

Who - Why - how - when - examples —> take your time to think about this

Interview guide METS training

Goal: mapping possible changes after METS workshop in attitude, thoughts and behaviours. Both workshop method and experiences with METS content.

General probes:
Do you know better what to do when this happens?
Do you know better how to deal with this?
Has the training helped you to deal with such situations? How?
How do you handle such situation?
How has this changed after the training?
Was this different before the training?
What was it like before the training?
What is it like now?
How did you feel about this before the METS? How do you feel about it now? Has your view on this changed after following the METS?

METS questions:
- How has learning about this topic influenced your life?
- What are the important things you will remember from this topic?
- How will this help you in your life? How/when/why/examples?

Who - Why - how - when - examples —> take your time to think about this

<table>
<tr>
<td>Introduction</td>
<td>- What did your life look like before the training? What does it look like now? Has anything changed, if so how?
- What were your expectations of the training? Have your expectations been met?
- What did you think about the training?
- What did you like? Why?
- Which parts of the training were useful?
- What did you not like? What could be improved?
- Is there anything special that changed for you because of the workshop? Skills-thoughts-behaviours-attitudes
- Are there any other big life events that happened during the workshop? (Positive and negative)</td>
</tr>
<tr>
<td colspan="2">The training consisted of seven themes, can you tell me a little bit about it?
1. Connectedness: Network & Aspects of trust
2. Hope: Positive attitude
3. Identity: Self Awareness & Strenghts
4. Meaning: Life Values
5. Empowerment: Coping & Acceptance
6. Recognition: Awareness and action plan & Culture and Rituals
7. Safety: Dealing with daily challenges & Stability</td>
</tr>
<tr>
<td>METS: connectedness</td>
<td>- How has learning about this topic influenced your life?
- What are the important things you will remember from this topic?
- How will this help you in your life? How/when/why/examples?
o First week: connectedness (network, trust and stigma).
Network:
▪ What was your social life like before the METS? How is social life now?
▪ Improvements?
▪ Establish more relationships?
▪ Better ideas on how to obtain and maintain new contacts?
▪ Has your awareness increased on the importance of asking and giving support?</td>
</tr>
</table>

Who - Why - how - when - examples —> take your time to think about this

	**Aspects of trust:**<br>▪ How did you feel about trusting others people before the METS?<br>▪ How do you feel about trusting others now?<br>▪ Has your view on trusting other people changed?<br>▪ Improve trust (in other people)?<br>▪ Improve connectedness (with other people)?<br>▪ For example by: Asking for help/support (practical, legal or physical)?<br><br>*When someone mentions **stigma**, further discuss this topic.*
METS: hope	○ Second week: hope (positive attitude and dreams). *When the following topics come up, discuss further (these are not interview questions):*<br>▪ (Positive) emotional state = your emotions/How you feel right now (with regards to your emotions)<br>▪ Sense of successful agency = the ability to act on your own behalf? Pathways? Changes after METS?<br>**Positive attitude:**<br>▪ Has it become easier for you to focus on positive things?<br>▪ Did you learn how to create hope and optimism?<br>▪ Did you learn how to implement positive thinking in your personal life?<br>▪ Do you feel more hopeful/optimistic than before?<br>▪ How hopeful did you feel before the training? How hopeful do you feel now? Did this change because of the training, if so how?
METS: identity	○ Third week: identity (self-awareness and strengths)<br>▪ How did you see yourself before the training? In terms of strengths.

	▪ How about after the training?<br>▪ Did you identify new strengths?<br>▪ (better) understanding of self? How do you notice? How will it help?<br>▪ Extended social groups with (better) understanding of identity?<br>▪ How do you experience your social roles? Discuss further: Less emphasis on refugee/homeless/illegal/etc.?<br>***Post traumatic growth****: if this topic comes up, discuss further.*
METS: meaning	o Fourth week: meaning (stress, challenges from the past and life values)<br><br>**Life Values:**<br>▪ Have you become more aware of your own values after the METS? Has this helped you? If so how?<br>▪ What kept you going/what motivated you before the METS? How is this now? Has this changed after the METS?<br>▪ What makes life worth living? Do you have another view on this after METS?
METS: empowerment	o Fifth week: empowerment (coping + acceptance)<br>▪ Understanding of empowerment = the process of becoming stronger and more confident especially in controlling one's life and claiming one's rights<br>▪ Gain control over own live?<br>▪ Coping styles: problem solving (actively seeking solutions), Venting emotions (expressing feelings), Seeking support (asking for help, comfort, advice, understanding), Thinking (analyzing problems, identifying causes), Disengagement (turning away)<br><br>**Coping:**

Who - Why - how - when - examples —> take your time to think about this

	▪ Are there certain strategies (coping styles) that you usually use to deal with problems?<br>▪ How did you deal with difficulties before the METS? can you give an example?<br>▪ How do you deal with difficulties now? Can you give an example?<br>▪ Has it become easier to deal with difficulties? Did you learn different/new ways of dealing with difficulties? (e.g. active problem solving, seeking social support, avoidance)<br>▪ Not so helpful (worsen problems) vs. helpful (help heal painful emotions, balanced look at situation and people, feel happy again)<br>▪ Did you learn about relaxation and grounding techniques? If so what did you think of them. Do you still use them?<br>**ABC framework:**<br>We know our thoughts influence our feelings.<br>- Do you think there is a difference between thoughts and feelings? Do you think they are related? In what way? Did you gain any new insights?<br><br>Do you feel you are more in control of your life after following the METS? Compare before/after METS
METS: Recognition	**Awareness and action plan:**<br>- Which problems/stressors do you experience in your daily life?/Are you aware of what your stressors are?<br>o How did you deal with these stressors before the METS?<br>o How do you deal with these stressors now?<br>o Do you approach problems differently since you followed the METS?<br><br>**Culture and rituals:**<br>- How did you incorporate rituals in your life before the METS?<br>- How do you incorporate rituals now?<br>- How has this changed after the METS?<br>- Is the use of rituals helpful for you? If so how?<br>- Has your view changed on this after the METS? If so how?

Who - Why - how - when - examples —> take your time to think about this

METS: Safety	**Safe place exercise**<br>- Before the METS, what did you do when you felt unsafe?<br>- What do you do when you feel unsafe now?<br>- Do you use the safe place exercise? Does it help you? If so, how?

Questionnaire versus evaluation	- What did you think about the questionnaires that you filled in?<br>- **Why do you think er asked you to fill them in?**<br>- How was it to fill these in? Difficult? Easy?<br>- **Was it useful for you**?<br>- Do you feel you understood how to do it properly?<br>- What about the explanations that were provided?<br>- How could it be made easier to fill them in?<br>- **Discrepancy: Evaluation very positive, questionnaire less. Why do you think this is the case**?
