# Supplementary material for: Undocumented Migrants’ Experiences of a Recovery-Oriented Group Intervention and Its Impact on Their Mental Well-Being: A Qualitative Study
Source: Int J Environ Res Public Health. 2025 Oct 23;22(11):1617. doi: 10.3390/ijerph22111617 (PMC12652161; doi:10.3390/ijerph22111617)
Supplement: Supplementary file 1 [file ijerph-22-01617-s001.zip › Topic list facilitators .docx]

**Topic list therapists brainstorm**

1. What is your professional background? (study, working experience)
2. How long/often have you been giving the METS?
3. What conditions (being with two people, at the office etc.) are important for you to ‘comfortably’ give the METS.
4. Did you feel prepared enough to give the METS?
5. How do you experience giving the METS?
6. What challenges did you experience when giving the METS?
   1. Which themes were difficult to convey to the participants? Why do you think this is?
7. What do you think the most useful part about the METS is?/ What do you think strengths of the METS are?
   1. What themes were easiest to convey to the participants? Why do you think this is?
8. Are there other things you like or don't like about the METS? What do you like about the METS?
9. What don’t you like about the METS?
10. How could the METS be improved?
11. What do you think about the METS being a group workshop?
12. How do you see participants at the beginning of the METS? how after? changes?
    1. Did you see any behavior or other changes in the participants during or after the workshop? If so, what type of change?
    2. also specifically mental health related?
13. What do you think are personal circumstances/ characteristics of participants that influence how well the message of the METS comes across?
14. Why do you think participants keep coming to the METS?
15. Why do you think some participants come irregularly to the METS?
16. Do you have an idea why some people stop coming to the METS?
17. Are there things you would like to add?
